# Human Brain Vasculature‐on‐a‐Chip Model Constructed With Microvessels Isolated From Cryopreserved Postmortem Human Brain Tissue

**DOI:** 10.1002/adhm.202504167

**Published:** 2026-01-12

**Authors:** Brian J. O'Grady, A. Scott McCall, Samuel Cullison, Daniel Chavarria, Matthew S. Schrag, Ethan S. Lippmann

**Affiliations:** ^1^ Department of Ophthalmology Vanderbilt University Medical Center Nashville Tennessee USA; ^2^ Department of Biomedical Engineering Vanderbilt University Nashville Tennessee USA; ^3^ Department of Pulmonary and Critical Care Vanderbilt University Medical Center Nashville Tennessee USA; ^4^ Department of Chemical and Biomolecular Engineering Vanderbilt University Nashville Tennessee USA; ^5^ Department of Neurology Vanderbilt University Medical Center Nashville Tennessee USA

**Keywords:** blood‐brain barrier, in vitro model, neurovascular unit, vasculature

## Abstract

Brain vasculature is a complex and heterogeneous structure that serves specialized roles in maintaining brain health and homeostasis. There is substantial interest in developing representative human models of the brain vasculature for drug screening and disease modeling applications. Many contemporary strategies have focused on culturing neurovascular cell types in hydrogels and microdevices, but it remains challenging to achieve anatomically relevant vascular structures that have similar function to their in vivo counterparts. Here, we present a strategy for isolating microvessels from cryopreserved human cortical tissue and culturing these vessels in a biomimetic gelatin‐based hydrogel contained in a microfluidic device. We provide histological evidence of arteriole and capillary architectures within hydrogels, as well as anastomosis to the hydrogel edges allowing lumen perfusion. In capillaries, we demonstrate restricted diffusion of a 10 kDa dextran, indicating intact passive blood‐brain barrier function. We anticipate this bona fide human brain vasculature‐on‐a‐chip will be useful for various biotechnology applications.

## Graphical abstract

This manuscript describes the cultivation of viable microvessels from cryopreserved human brain tissue. When embedded in hydrogels and cultured in microfluidic devices, these microvessels exhibit complex architectures reminiscent of arterioles and capillaries, can be perfused, and display intact barrier function. Collectively, these results demonstrate a new model of human brain vasculature suitable for biological investigations.

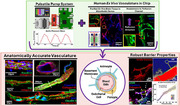

## 1 Introduction

The human brain's intricate physiology is supported by a complex network of vasculature spanning a heterogeneous arteriovenous axis [1]. The brain vasculature carries out diverse functions and possess unique cellular and molecular signatures relative to peripheral vasculature. For example, arterioles contribute to blood flow regulation through neurovascular coupling mechanisms [2], while capillaries serve as the site of material exchange between the bloodstream and parenchyma, as controlled by the properties of the blood‐brain barrier (BBB) [3]. Anatomically, vessels in the brain are lined with highly specialized endothelial cells and ensheathed by mural cells within a collagen‐rich basement membrane. The outer edge of the vessels is surrounded by astrocyte end feet, collectively creating the intricate 3D structure of the neurovascular unit (NVU). All of these cells actively communicate to ensure proper neurovascular function, and in various disease states, these functions are compromised [4]. As such, there is significant interest in developing more physiologically relevant in vitro models of the human cerebrovasculature to better understand disease mechanisms and develop therapeutic intervention strategies for both acute insults and chronic degenerative conditions.

Many strategies have been developed to recapitulate the 3D architecture of the NVU in vitro [5, 6, 7]. These strategies generally rely on the self‐assembly capacity of endothelial, mural, and glial cells, which are typically sourced from primary material or differentiated from human pluripotent stem cells (hPSCs). In general, cells are cultured in hydrogels contained within well plates or microfluidic devices, the latter of which permits assessments of vascular perfusion and measurements of barrier properties. Culture in hydrogels allows growth or dynamic remodeling as neurovascular cells assemble into their desired structures [8, 9]. Other models have relied on direct placement of cells in specific compartments, for example lining prefabricated channels in hydrogels with endothelial and mural cells, while astrocytes or other neural cells are cultured within the hydrogel [10, 11, 12]. While all of these models have achieved some structural features of cerebral vasculature, they generally lack the organized architectures seen in vivo, which would ultimately be more desirable for studying the intimate communication between cell types in the NVU across different levels of the vascular tree.

**FIGURE 1:**
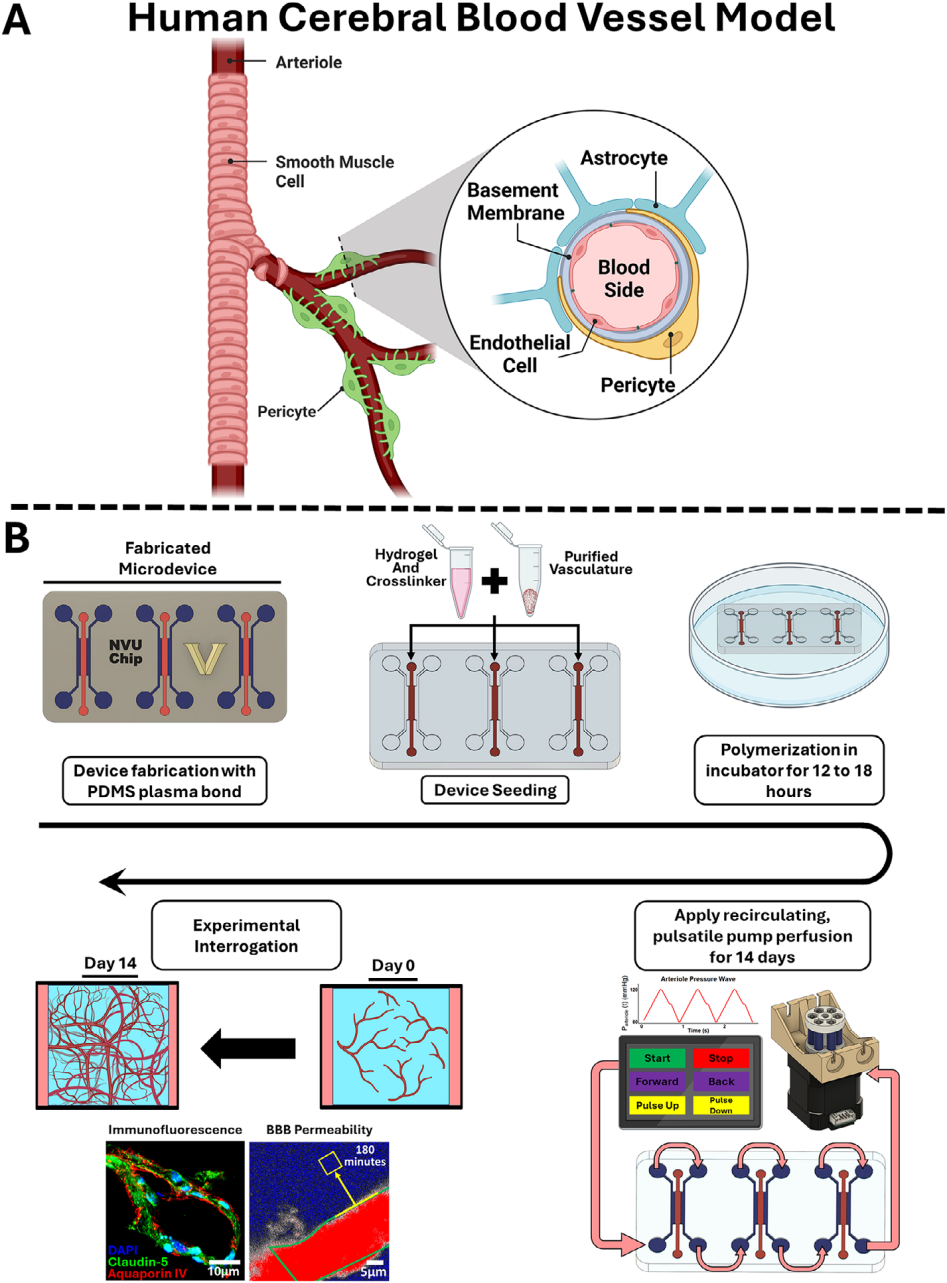
Overview of the ex vivo human vasculature‐on‐a‐chip model. A) A detailed graphic representation of the human neurovascular unit (NVU), highlighting the spatial arrangement of lumenized endothelial cells surrounded by a basement membrane, pericytes, and astrocyte endfeet. B) Timeline of the NVU model fabrication and experimentation process. Custom microdevices are fabricated and seeded with purified cerebral blood vessels in an enzymatically crosslinked hydrogel. The hydrogel undergoes polymerization in an incubator for 12 to 18 h, and then a recirculating pulsatile pump perfuses the microdevice for 14 d. During this period, the vasculature grows and develops within the hydrogel matrix. On day 14, various analyses are performed to assess the functionality and representativeness of the NVU model.

Here, to better mimic endogenous cerebrovasculature architectures in vitro, we present a human brain vasculature‐on‐a‐chip model that is fabricated using microvessels enriched from postmortem, cryopreserved human cortical tissue (Figure 1). We developed an enzyme‐free method to collect and purify human microvessels, which helps maintain native vessel architectures. Then, rather than relying on assembly of individual cell types into the desired neurovascular structures, these pre‐existing vessels are embedded in a peptide‐functionalized gelatin‐based hydrogel within a custom microfluidic chip under conditions that promote vascular growth. Using immunohistology and live imaging, we demonstrate that these vessels possess native architectures, are perfusable, and retain cellular identities reminiscent of cerebral vasculature. Excitingly, interconnected arterioles and capillaries are reliably detected in the hydrogels, and the capillaries demonstrate robust passive barrier function as indicated by a tracer extravasation assay. Overall, we anticipate this model system will open new avenues for understanding neurovascular function in health and disease.

## 2 Methods

### 2.1 Microfluidic Chip Design, Fabrication, and 3D Printed Tools

The master mold for the microfluidic chips were fabricated either using a stereolithographic (SLA) 3D printer (Formlabs 3) or a Haas CM‐1 CNC. Printed molds were fabricated using Formlabs Black resin v3. The molds were extensively washed in 100% isopropanol alcohol to remove all uncrosslinked monomers and photocrosslinking agents. The molds were then extensively washed with ultra‐pure water and allowed to air dry in front of a fan for 30 min. The washed molds were then placed in a Formlabs Cure chamber and exposed to ultraviolet light for 10 min. All prints were then treated with parylene as previously described [13], which allows them to serve as master molds for elastomers. In some cases, 6061 aluminum stock (9146T69; McMaster‐Carr) was milled on the CNC to create master molds. Additional information (including CAD files, tools used, generated paths, feeds and speeds, etc.) are available on GitHub.

To create microdevices, PDMS (Sylgard 182; Dow Corning) was poured onto the 3D printed and/or CNC machined master molds, and the molds were then placed in a vacuum chamber to remove all bubbles and subsequently heat‐cured at 80°C for 30 min. After heat‐curing, the solidified PDMS was carefully removed from the master molds and a biopsy punch (1.5 mm) was used to create inlets and outlets for the hydrogel injection channel and the media perfusion channels. Using scotch tape, debris was removed from the surface of the PDMS, and then microdevices were plasma bonded to a precleaned microscope glass coverslip (Ted Pella 260461‐100). Prior to experimentation, microdevices were sterilized using an autoclave. STL files and parts lists for all microdevices are available on GitHub.

### 2.2 Pump Perfusion System

A customized pump perfusion system was modified from a previous system [14]. Briefly, the system uses a Raspberry Pi 4, Raspberry Pi touchscreen, Easydriver, and a stepper motor with a 12v power supply. A low‐cost, custom printed circuit board was designed and fabricated to integrate the electronics for this system. Custom code was written to generate desired pulse waves, where the software allows the user to control the direction of the fluid flow, duration to the systolic amplitude, pulse duration, time in between pulses, duration of the dicrotic notch, and control over the timing of the dicrotic notch. Flow profiles generated by the pump were recorded using a Sensirion liquid flow sensor that was placed immediately prior to the inlet of the microdevice, and the frequency of the pulse wave was modeled after a 60 Hz cardiac cycle. The total dead volume of the tubing and pump circuit was measured at approximately 1.2 mL, while the fluid volume within the microfluidic chip is approximately 50–75 µL. Schematic designs, CAD files, device assembly, design rule checking list, all code, design files, bill of materials, and assembly videos are available on Github.

### 2.3 GelCad Biomaterial Synthesis

GelCad biomaterial was synthesized as previously described with minor modifications [15, 16]. Briefly, Type A porcine skin gelatin (Sigma) was reconstituted in triethanolamine (TEOA; Sigma) to create a 10% w/v solution and stirred at 37°C for 2 h until fully dissolved. The pH of the solution was then adjusted to 8.0–8.5 by adding 1.0 m HCl or 1.0 m NaOH. An intermediate reaction was used to prepare the peptides for binding. 80 mg of ethylcarbodiimide hydrochloride (EDC; Thermo Fisher) and 120 mg of n‐hydroxysuccinimide (NHS; Thermo Fisher) were mixed with 80 mg of peptide. The combined solids were dissolved in 10 mL of N‐N‐dimethyformamide (DMF) (28 mg mL^−1^), and 15 mL of PBS (19 mg mL^−1^) at 37°C for 1 h. The peptide intermediate was then added dropwise to the gelatin solution and reacted for 4 h at 37°C with constant stirring. Next, the reaction was quenched by raising the pH to 8 using sodium bicarbonate (5 m). The final solutions were dialyzed using a Slide‐A‐Lyzer Dialysis Flask (ThermoFisher 87761; 3.5 kDA MW cuttoff) with ultrapure water. After filtration, the biomaterial solutions were frozen, lyophilized, and pulverized for long term storage.

### 2.4 Complete Media Preparation

Heat stable basic fibroblast growth factor (bFGF; Gibco PHG0367) was reconstituted by adding 1 mL of PBS to a 5 µg vial. Epidermal growth factor (EGF; PeproTech AF‐100‐15‐1MG) was reconstituted by adding 1 mL of PBS to a 1 mg vial. Fibronectin (ThermoFisher 33016015) was reconstituted by adding 5 mL of PBS to a 5 mg vial. Vascular endothelial growth factor (VEGF, PeproTech 100–20) was reconstituted by adding 1 mL of PBS to a 10 µg vial. Thymosin Beta‐4 (TB4, PeproTech 140‐14) was reconstituted by adding 1 mL of PBS to a 20 µg vial. All components were aliquoted and stored at ‐20°C. Complete media was prepared by combining 17.75 mL of human endothelial serum‐free medium (hESFM; ThermoFisher 1111044) with 1 mL reconstituted bFGF, 10 µL reconstituted EGF, 500 µL reconstituted fibronectin, 500 µL reconstituted VEGF, and 250 µL reconstituted TB4, creating a final volume of 20 mL.

### 2.5 Microfluidic Chip Design

The microfluidic chip features a central channel measuring 1.532 mm in width and 0.152 mm in depth, flanked by two larger perfusion channels, each 1.047 mm in width. This configuration accommodates large fluid volumes. The chip's pillarless design eliminates the traditional structural supports that can lead to hydrogel spillover into perfusion channels—a common issue in larger‐featured devices. This design enhances the reproducibility of the experimental setup by preventing asymmetry in the chips, which could otherwise compromise experimental outcomes. Furthermore, the central channel's extended length offers enhanced control during the hydrogel injection process. This design feature further ensures that the hydrogel remains confined to the designated area, minimizing the risk of leakage into adjacent perfusion channels.

### 2.6 Microfluidic Chip Holder for Hydrogel Injection

A custom‐designed microfluidic chip holder, depicted in Figure S1, was developed to complement the chip design. This holder positions the chip at a precise angle during hydrogel injection, which helps fill the central channel without spillage of hydrogel into the perfusion channels.

### 2.7 Collection of Human Brain Tissue and Preparation for Microvessel Enrichment

Most of the data in this manuscript were collected using human brain tissue obtained at autopsy from a 73‐year‐old male patient who presented with mild to moderate generalized atrophy, severe Alzheimer's disease (Braak stage IV, Thal stage 5, A3, B3, C3), moderate cerebral amyloid angiopathy (CAA), and mild atherosclerosis. There was no evidence of Lewy bodies or other tauopathies, and the post‐mortem interval was 8 h. A second donor—78‐year‐old male patient with presumed CAA and a post‐mortem interval of 16 h—was used for validation of select outcomes. Tissue collection was conducted under an IRB‐approved protocol at Vanderbilt University Medical Center (#180287). After manually separating cortical grey matter from white matter, tissue was cut into ≈1 cm^3^ pieces and placed into cryovials that were subsequently filled with BrainBits Hibernate A media containing 10% DMSO. Cryovials were slowly cooled in a Nalgene Mr. Frosty container overnight in a ‐80°C freezer. The following day, cryovials were placed in liquid nitrogen for long‐term storage. For experiments, a single cryovial was removed from liquid nitrogen and slowly brought up to room temperature in a liquid or bead bath. Once 70% thawed, the vial was transferred to a sterile cell culture hood and freezing media was aspirated. Then, the brain tissue was transferred to a 2 mL ultralow attachment vial. The vial was filled with 1 mL of cold hESFM medium and the tissue was homogenized with tweezers for 1 min. This step was followed by subsequent manual homogenization with hand pipettes, first with albumin‐coated 1 mL pipette tips and then 200‐µL albumin‐coated tips. Finally, the homogenized brain tissue was microcentrifuged at 200 rcf for 5 min, and the supernatant was aspirated and discarded. The vial was then filled with cold hESFM media to transfer the tissue to the filtration device.

### 2.8 Filtration Device Design and Microvessel Enrichment

A custom 3D printed filtration device was developed to enrich microvessels from homogenized tissue. Designs for the filtration device are available on Github. As previously described, the 3D printed parts are coated with parylene before use to ensure biocompatibility. The device contains three components: an upper cap with threads and a lip, a lower cap, and a laser cut nylon mesh (100 µm filter size) in between. The nylon mesh fits in the center of the bottom cap, and then the top cap is screwed onto the lower cap. A gasket o‐ring (McMaster Carr 90025k364) is placed underneath the lip on the top cap to act as a seal. The entire filtration device is then placed in a 150‐mL side arm Erlenmeyer flask (Fisher 10‐180D). The side arm is then attached to a pipette aspirator. Using an albumin‐coated pipette tip, the homogenized brain tissue is transferred from an ultra‐low attachment vial onto the mesh filter. Sterile PBS is then poured onto the mesh while slightly pulling pressure with the pipette aspirator. This strategy pulls the PBS down through the filter to carry away dead or singularized cells, leaving behind enriched microvessels. After this step, the mesh filter is removed and the microvessels are gently scraped into an ultra‐low attachment six‐well plate containing cold hESFM for additional washing. Next, the microvessels are transferred to a 2‐mL ultra‐low attachment vial and centrifuged at 400 rcf for 5 min. Last, the supernatant is aspirated and the microvessels are resuspended in 250 µL of 10% GelCad precursor solution (w/v in complete media) containing 15 µL microbial transglutaminase (Modernist Pantry Moo Gloo TI 1203‐50).

### 2.9 Microvessel Culture in Microfluidic Chips

After microvessels have been isolated and resuspended in GelCad precursor solution with transglutaminase, microdevices are prepared for injection. First, microfluidic chips are placed in the 3D printed holder with the long part of the central channel face down (Figure S1). Using the PDMS fragments originally removed via biopsy punch, the flanking perfusion channels are temporarily plugged at the inlet and outlet, which helps prevent hydrogel from vacating the central channel after injection. An electronic autopipette is then used to inject the microvessel solution into the central channel. The autopipette facilitates a uniform, slow injection that is continued until the microvessel solution reaches the port on the opposite side of the device. After injection, microdevices are carefully removed from the chip holder and placed into an incubator to crosslink for a minimum of 4 h and a maximum of 18 h. After hydrogel crosslinking was confirmed, the microfluidic chip was connected to the pump perfusion system. Perfusion experiments were run for 14 d, unless otherwise specified.

### 2.10 Immunohistology

For fixation, the hydrogels from the microfluidic chips were cut out of the PDMS as previously described [17]. The hydrogels were then submerged in 4% paraformaldehyde for 30 s. The fixed hydrogels were then washed with PBS 3 times at 15 min intervals. Next, the fixed hydrogels were permeabilized in PBS containing 0.1% Triton X‐100, 0.1% sodium azide, and 5% normal goat serum overnight at 4°C on a rocker. Hydrogels were subsequently washed 5 times with PBS and then blocked again with PBS containing 0.05% Tween‐20 and 5% normal donkey serum for 40–60 min. Hydrogels were then incubated with primary conjugated antibodies overnight at 4°C on a rocker. All antibodies were used at a concentration of 1:1000. The antibodies used were as followed: NG2‐Alexa Fluor 647 (Abcam; ab183929), Aquaporin 4‐Alexa Fluor 488 (Abcam; ab284135), PODXL‐Alexa Fluor 568 (Abcam; ab211225), Beta‐III Tubulin‐Alexa Fluor 555 (Abcam; ab202519), Ki67‐Alexa Fluor 488 (Abcam; ab197234), PECAM1‐Alexa Fluor 488 (Cell Signaling; 42777), Alpha‐Smooth Muscle Actin eFluor 660 (Thermofisher; 50‐9760‐82), Claudin‐5‐Alexa Fluor 488 (Thermofisher; 352588), Lectin DyLight 488 (L32470), Actin (Phalloidin)‐ Alexa Fluor 555 (Thermofisher A34055), Collagen IV‐Alexa Fluor 647 (Thermofisher; 51‐9871‐82), GFAP‐Alexa Fluor 488 (Thermofisher; 53‐9892‐82). Fluorescent images of immunostained structures were obtained using a Zeiss LSM 880 confocal microscope at ×10 and ×20 magnifications. The hydrogel was scanned at various focal planes, spanning from z = 0 to 100 µm, with intervals of 8–10 µm. For the purpose of generating a 3D representation of the images, the 3D Viewer plugin within ImageJ software was employed.

### 2.11 Measurement of Permeability in Capillaries

The permeability of a 10 kDa dextran labeled with Texas Red was determined by analyzing the diffusion from the vasculature into the surrounding hydrogel matrix. For each permeability assay, a separate microfluidic chip was used, constituting a biological replicate. To locate capillaries for permeability measurements, we first identified vessels with a diameter range of 5–10 µm. A secondary criteria was that each selected capillary needed to be at least 50 µm away from any other vascular structure to minimize external influences on the diffusion measurement. Fluorescent microscopy was used to capture the initial and final distribution of the dextran within a specified field of view (FOV). The initial FOV was defined as 244.03 µm by 193.99 µm, with image resolution of 1385 by 1101 pixels. Fluorescence intensity was quantified from a 10×10 pixel box within this FOV, averaged, and recorded as a single value corresponding to discrete time points over a duration of 3 h. Red pixel quantification was carried out on the images, with “red” defined by a custom threshold that discriminates the dextran fluorescence from the background. A pixel was classified as “red” if its red channel intensity exceeded a predefined minimum value and was higher than that of the green and blue channels. The total count of ‘red’ pixels represented the dextran‐laden area at each time point.

The permeability coefficient was calculated as follows:

1. Area per pixel: The area represented by each pixel (A_pixel_) was derived by dividing the FOV area by the total pixel count in the image.
$$A_{\mathrm{pixel}}=\frac{\mathrm{FO}V_{\mathrm{area}}}{\mathrm{Total}\;\mathrm{pixel}\;\mathrm{count}}$$
2. Dextran concentration: The initial concentration of dextran (*C*_0_) was set at 10 mm. This concentration was used to determine the molar quantity of dextran within the red pixel area at the initial and final time points.
$$C_{0}=10\;\mathrm{mm}$$
3. Molar flux (*J*): The flux was calculated using the change in molar quantity of dextran (Δ𝑛) over the diffusion area (𝐴_diffusion_), normalized by the experiment duration (*t*).
$$J=\frac{\Delta n}{A_{\mathrm{diffusion}}\times t}$$
4. Permeability coefficient (*P*): Fick's first law was applied to define *P*, with *J* divided by the concentration gradient across the capillary (Δ𝐶), which was approximated by the initial dextran concentration (*C*_0_).
$$P=\frac{J}{\Delta C}$$

The change in the red pixel count between the initial and final images was used to calculate Δ𝑛, while 𝐴_diffusion_ was assumed to be the total red pixel area at the initial time point. *P* was initially obtained in µm min^−1^ and then converted to cm s^−1^ for standardization:

$$P_{\mathrm{cm}\;s-1}=P_{\mu m\;\min-1}\;\times \left(\frac{10^{-4}\mathrm{cm}}{1\;\mu m}\right)\;\times \left(\frac{1\;\min}{60\;s}\right)$$

## 3 Results

### 3.1 Vessel Purification and Assessments of Structural Integrity

**FIGURE 2:**
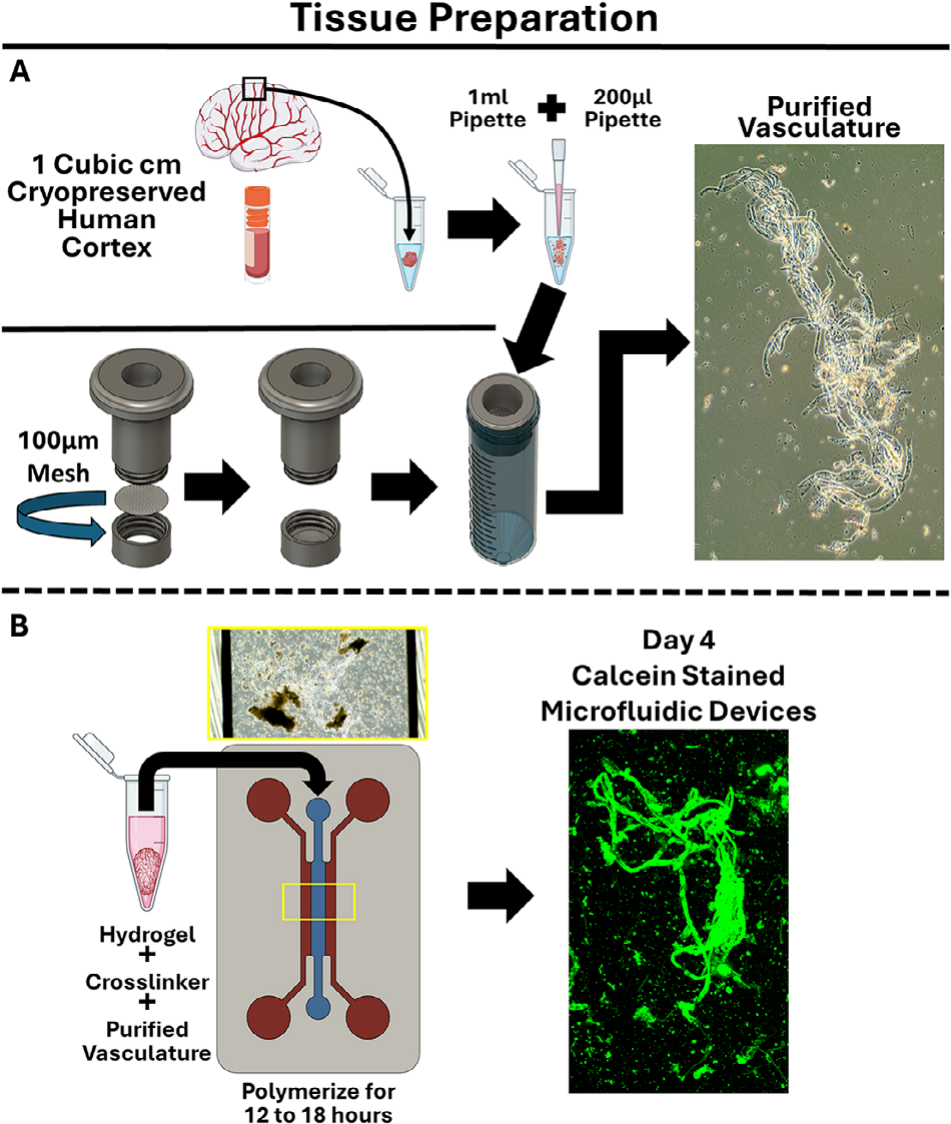
Strategy for obtaining and culturing purified vasculature from cryopreserved human cortical tissue. A) ≈1 cm^3^ piece of cryopreserved human cortical brain tissue is transferred to a 2 mL ultra‐low attachment conical tube and homogenized using hand pipettes. Vessels are purified from the dissociated tissue using a custom filtration device equipped with a 100 µm mesh filter. A representative brightfield image of isolated vessels is shown on the right. B) Purified vessels are reconstituted in a hydrogel precursor solution mixed with an enzymatic crosslinker. The mixture is gently injected into the center chamber of the custom microfluidic device, allowing the hydrogel to crosslink overnight. A representative image of calcein‐stained tissue is shown on day 4 of culture within the microdevice, showcasing high viability of the embedded microvessels.

Extracting intact microvasculature from human tissues is a challenging endeavor due to small size, relative scarcity within tissue, intricate architecture, and the need to preserve cellular viability. Traditional methods often rely on enzymatic digestion, which can lead to partial degradation or loss of critical components such as endothelial cell tight junctions and the arrangement of other cells in and around the vessels, thus compromising the structural integrity of the vessels. To address this challenge, we developed an enzyme‐free protocol for the extraction of human microvessels from cryopreserved postmortem cortical tissue, aimed at preserving the structural integrity of the microvascular networks (Figure 2A). After isolation, the enriched microvessels were embedded in a hydrogel within a perfused microfluidic device for 4 d before initial analysis—the hydrogel and microfluidic device are described in more depth below. After 4 d of culture, the microvessels had high viability as seen by calcein staining (Figure 2B). To validate the structural integrity and cellular composition of the enriched vasculature, we performed extensive immunostaining, with the results presented in Figure S2. The staining results reveal a comprehensive picture of the preserved vessel architecture. We provide evidence of both capillary and arteriole structures based on vessel size and the presence of NG2+ and ⍺‐SMA+ mural cells around PECAM‐1+ and claudin‐5+ endothelial cells. Positive aquaporin‐4 (AQP4) signal around vessel structures suggests the presence of astrocyte end feet, and immunostaining for PODXL, a sialoglycoprotein found on the luminal side of brain endothelial cells [18], indicates maintenance of the glycocalyx. We could detect βIII‐tubulin+ neurons that are likely remnants of the purification process, but we did not assay for their survival or include them in any downstream analyses. Positive immunostaining for Ki‐67 suggests vessel growth occurs after embedding in the hydrogel. Using a second donor, we validated viability of isolated microvessels with calcein staining and maintenance of vascular architecture by PECAM‐1 immunolabeling (Figure S3). In this donor, we also further confirmed the presence of astrocytic cell bodies and processes extending around some of the vessels by immunolabeling for GFAP (Figure S3). Overall, these data support our claims that the enzyme‐free extraction method effectively preserves the structural integrity of isolated human microvessels and enables their culture within hydrogels, with secondary evidence that other neural cell types may be spuriously present.

### 3.2 Pump Perfusion System

**FIGURE 3:**
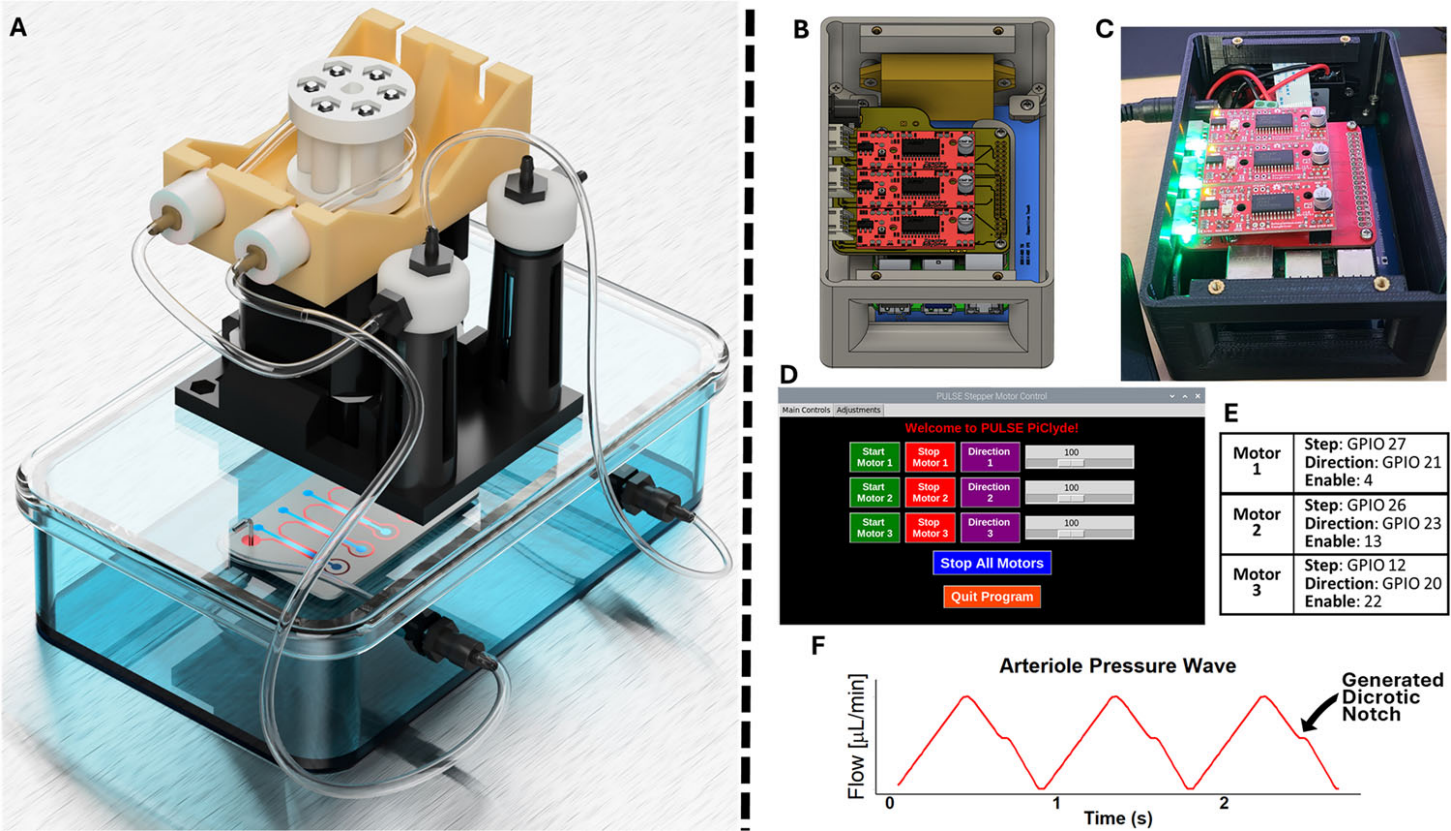
Custom pump perfusion system. A) Representation of the experimental system, showing the stepper motor constituting the pump, the container holding the microfluidic device, the device itself, and the fluid reservoir. B) CAD design of the custom pump perfusion system, illustrating the EasyDriver motor controllers, custom PCB, Raspberry Pi, touchscreen, and housing unit. C) Actual built design of the CAD model, showcasing the assembled components. D) Custom graphical user interface developed to control three motors individually, providing precise control over each motor's operation. E) Stepper motor with corresponding step, direction, and enable pins, essential for controlling the motor's movements. F) Pulse wave generated by the pump perfusion system, as recorded using a Sensirion flow sensor at the inlet of the microdevice. The pulse wave includes a dicrotic notch, indicated on the graph, which is present in arterial pulse waves.

After confirming structural integrity of isolated human microvessels with hydrogels, we pivoted towards their extended culture. To achieve constant perfusion of the microdevices, we developed a custom pump perfusion system capable of generating pulse waves to the microfluidic systems and allow meticulous control over pulse rate, pressure wave amplitude, pulse frequency, dicrotic notch timing, and pulse width modulation (Figure 3). We further built open‐source hardware and designed a custom graphical user interface to simplify the creation of pulse waves tailored to specific experimental needs. The development of this system was motivated by extensive research establishing that the circumferential mechanical strain of arteries and arterioles is a crucial element in vascular growth and plays a critical role in regulating vascular structures and function [19, 20]. However, to our knowledge, there were no commercially available pump perfusion systems able to mimic the endogenous arterial pressure wave, including the characteristic dicrotic notch produced by our setup. Here, it is important to note that we did not assess whether this particular flow profile was critical to vessel development and establishment of passive barrier function as described in the following sections. However, the customizable pump and interface provide an avenue for future studies to explore the importance of these parameters and should be a valuable tool for the research community.

### 3.3 Vascularization of Hydrogel‐Laden Microfluidic Devices Under Constant Perfusion

**FIGURE 4:**
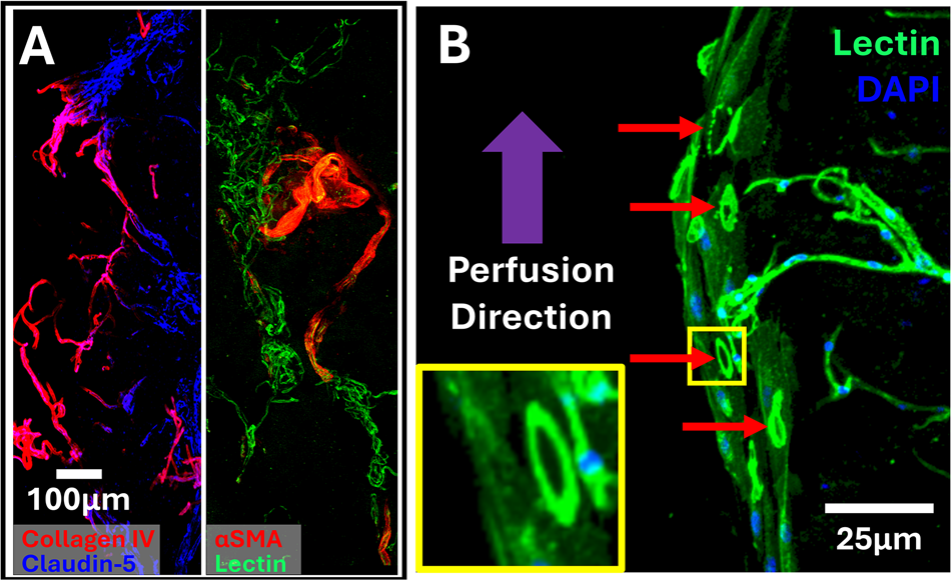
Vascularization of hydrogels within the microfluidic device after 14 d of culture. A) Representative images of collagen IV and claudin‐5 (left panel), and ⍺SMA and lectin (right panel). B) Representative images of lectin+ vessels at the interface of the hydrogel and the perfusion channel. The red arrows and yellow inset highlight the formation of lumenized structures along the edge of the hydrogel.

Next, we proceeded with extended culture of the human microvessels within hydrogel‐laden microfluidic devices. Similar to the condition for the short culture times described in Figure 2, we used a gelatin‐based hydrogel functionalized with a biomimetic peptide derived from the homophilic cell adhesion epitope of N‐cadherin (“GelCad”) to promote cell integration in the 3D environment. Previous work has shown that the conjugation of N‐cadherin peptides with biomimetic hydrogels are largely beneficial for cellular integration in 3D environments [15, 16, 21, 22, 23, 24]. Hence, we anticipated that GelCad hydrogels would support survival and outgrowth of human microvessels. To this end, we cultured microvessels for 14 d in the microfluidic devices under constant recirculating perfusion. Immunostaining at the endpoint revealed robust networks of claudin‐5+/collagen IV+ vessels (Figure 4A). Visualization of lectin and ⍺SMA further revealed the presence of ⍺SMA+ arterioles connected to ⍺SMA‐/lectin+ capillary‐like structures (Figure 4A). Imaging of the lectin+ vessels near the edge of the hydrogel demonstrated anastomosis with the perfusion channel (Figure 4B), indicative of fluid entry into the vessels from the perfusion channel, which we explored later.

**FIGURE 5:**
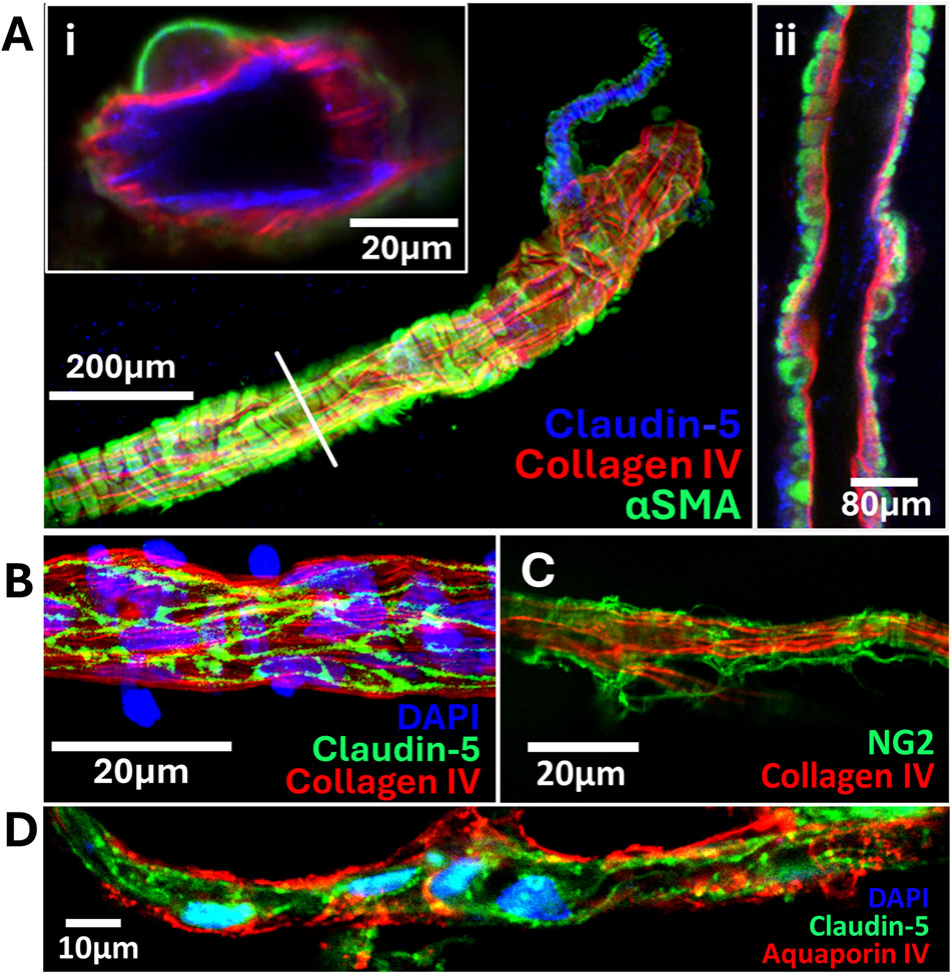
Anatomical arrangement of arterioles and capillaries within the microfluidic device after 14 d of culture. A) Representative image of an arteriole identified by concentric rings of smooth muscle cells. Insets show different cross‐sectional images highlighting the correct concentric anatomical arrangement of the arterioles. B, C) Representative images of capillaries showing intact tight junctions in endothelial cells (panel B) and pericytes wrapping around the vessel (panel C). D) Representative image of AQP4+ endfeet surrounding a vessel. Each panel is from an individual biological replicate (separately thawed vial of tissue; *N* = 4).

More extensive immunostaining at the 14 d endpoint confirmed robust vessel architectures. We were able to detect arterioles with in vivo‐like architectures consisting of concentric rings of smooth muscle cells wrapping around claudin‐5+ endothelial cells, separated by a collagen IV+ basement membrane (Figure 5A). Cross‐sectional images showcased the patent lumens of these arteriole structures (Figure 5A‐i, ii). We could further detect conjoining claudin‐5 tight junctions within the collagen IV+ structures and NG2+ pericytes wrapping around the outside of capillary‐sized vessels (Figure 5B,C). AQP4 signal remained detectable on the outside of vessels, again suggesting the presence of astrocyte endfeet (Figure 5D). Collectively, these results demonstrate that the human microvessels can be effectively cultured within hydrogel‐laden microfluidic devices for extended time periods and maintain vessel architectures mimicking native brain tissue.

### 3.4 Evaluation of Engineered Microvessel Perfusion and BBB Permeability

**FIGURE 6:**
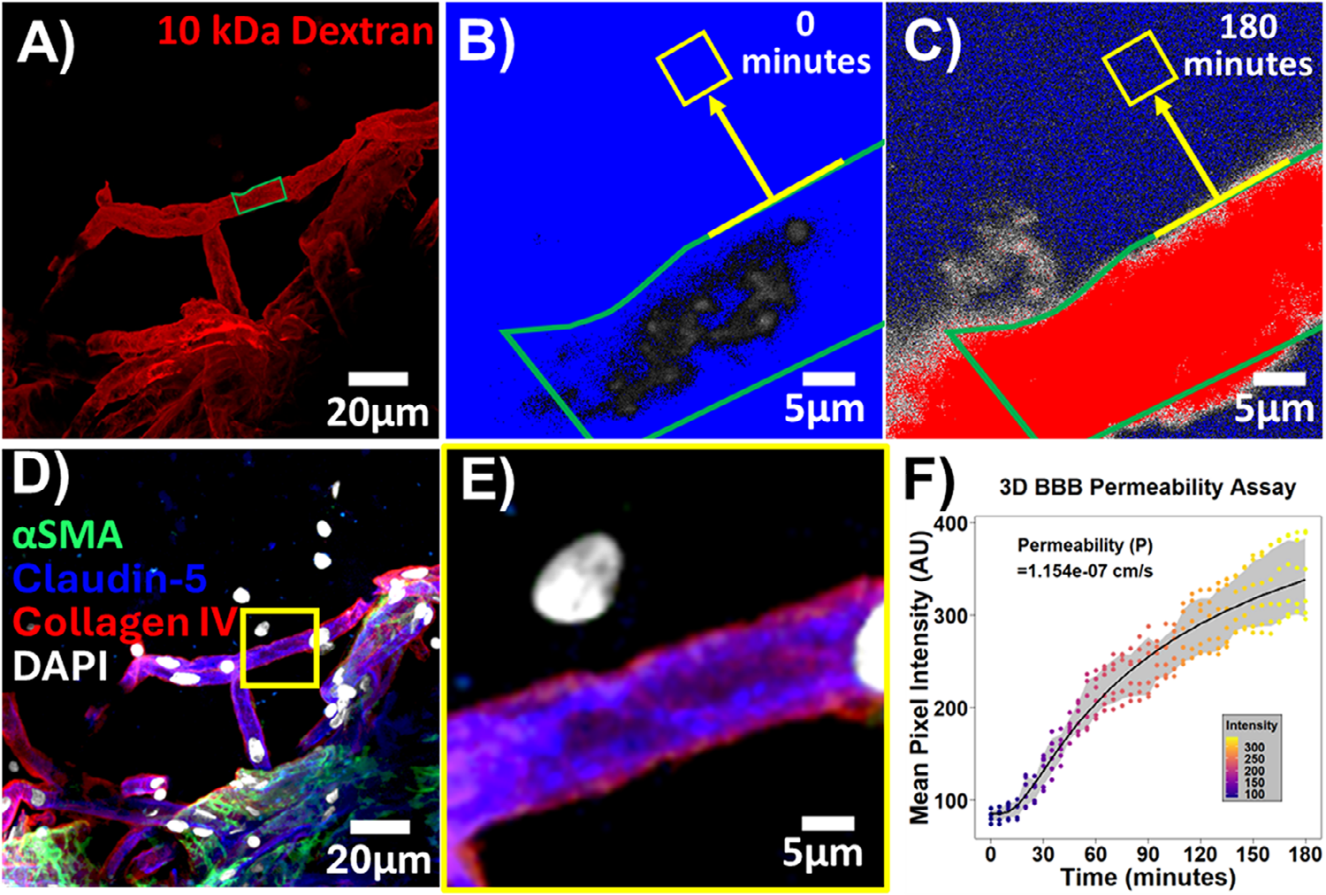
BBB permeability assay. A) Representative image of Texas Red‐labeled 10 kDa dextran perfusion through vessels at the day 14 time point. B,C) Example baseline image (time = 0 min) and final image (time = 180 min) for dextran extravasation. The yellow box indicates the specific region of interest, taken 10 µm away from the capillary, for the full time‐lapse imaging. D,E) Immunofluorescence labeling of the fixed vessels after the permeability assay to identify and characterize the cellular components of the vasculature. The yellow inset highlights the region where the time‐lapse imaging was performed. Comprehensive labeling confirms the assay was performed in a capillary lacking ⍺SMA coverage. F) Quantitative analysis of BBB permeability based on the change in Texas Red signal over time. Measurements were collected every 5 min, and each dot at the respective time point represents an individual biological replicate (separately thawed vial of tissue; *N* = 6 independent microdevices and permeability measurements). The calculated permeability coefficient (*P*) is 1.154e‐07 cm s^−1^, and the shaded region represents the standard error of the mean (SEM) from the biological replicates.

To evaluate barrier function of the microvessels in our system, we utilized a 10 kDa dextran conjugated to Texas Red dye. At our standard 14‐day timepoint, we introduced the dextran into the flanking perfusion chambers, where it readily entered anastomosed vessel lumens indicating their ability to be perfused (Figure 6A). Time‐lapse confocal microscopy was then used to assess dextran extravasation from the lumen of individual capillaries to the extracellular matrix. The baseline image taken at 0 min (Figure 6B) shows the initial distribution of dextran. After 180 min (Figure 6C), the dextran remained largely confined within the vascular lumen, with only minor diffusion into the surrounding tissue. At the end of the assay, immunostaining was used to confirm capillary integrity by the presence of claudin‐5+ endothelial cells and a collagen IV+ basement membrane lacking coverage by smooth muscle cells (Figure 6D,E). The quantitative analysis, presented in Figure 6F, showed a permeability coefficient for Dextran was calculated to be 1.154×10^−7^ cm s^−1^, a value consistent with those measured in other BBB models [8, 12, 17].

## 4 Discussion

In this study, we demonstrate the ability to engineer lumen‐perfusable human cerebrovasculature within a microfluidic device. To our knowledge, there are currently no methods that have effectively grown ex vivo vasculature and maintained the native vessel architectures seen in the brain in vivo. Our technology therefore addresses obstacles in replicating these complex structures in vitro. To overcome challenges associated with promoting self‐assembly of individual neurovascular cell types into vessel‐like structures, we employed the novel approach of culturing purified human cortical microvessels in a biomimetic hydrogel connected to a custom perfusion system. This system promoted growth and anastomosis of vessels to the edges of the hydrogels, and we could detect perfusable vessels with both arteriole and capillary architectures that were interconnected in the hydrogel.

Most models of the cerebrovasculature have relied on microfluidic device designs that enable culture of brain endothelial cells on porous planar substrates or on the edges of hydrogels, which can be further interfaced with a separate chamber harboring neural or glial cells [10, 25, 26, 27, 28, 29, 30]. In contrast, engineered models of 3D vascular networks at the level of capillaries and arterioles generally rely on self‐assembly of individually prepared cell types into representative vascular structures within hydrogels. Some notable model examples include the assembly of endothelial cells, pericytes, and astrocytes into perfusable vessel structures in microfluidic devices [8, 9, 17], and the assembly of endothelial cells, mural cells, and astrocytes under static conditions [31]. However, these self‐assembled models have noted structural deficiencies that our use of primary human microvessels can overcome. As one example, to our knowledge, no prior models constructed with individual cells have demonstrated concentric rings of smooth muscle wrapping around lumenized endothelial cells, representing bona fide arteriole structures. Generally, it has also been challenging to achieve vessel diameters representing capillary formation (5–10 µm), and recent advancements that can achieve endothelialized structures of this size still lack pericyte coverage [32]. Astrocytes, while present in some of these systems, do not typically extend their endfeet along the entirety of the vessel surface [12], as seen in vivo. Further, arteriole and capillary vessels have not been generated within the same hydrogel, nor with interconnected perfusion in the same vascular network. We provide evidence that each of these features can be achieved with primary human microvessels cultured in our biomimetic hydrogel. We believe that a critical aspect of our approach is the ability to preserve microvasculature integrity and cell viability through an enzyme‐free extraction method. This step maintains cerebrovascular architecture within the microfluidic platform as a starting template for vascular development. The preservation of these structures is likely vital for studying cerebrovascular function, where cell–cell and cell–matrix interactions are more likely to mimic native in vivo conditions.

After replicating the structural characteristics of the cerebral blood vessels, we assessed permeability of capillaries using a dye extravasation assay. Unlike many models that fail to achieve robust barrier integrity, our ex vivo model maintains a low permeability coefficient comparable to in vivo conditions [33]. These values are consistent with permeability estimates derived from MRI‐based assessments of BBB integrity in the human brain, which similar report restricted diffusion of low molecular weight solutes under physiological conditions [34, 35, 36]. Comparisons between planar versus more lumenized in vitro BBB models, particularly those fabricated from iPSCs, suggest 3D architecture is important for maintaining robust BBB function [11, 26, 29]. In our system, the close apposition of pericytes to the brain endothelial cells likely reinforces the observed integrity of the endothelial barrier [37, 38], although this claim must be supported by follow‐up work assessing the molecular signatures of cells in the model and other aspects of BBB function such as transporter activity.

Beyond cellular organization, a unique feature of our model is the application of a hemodynamic profile within the microdevices that mimics the arterial pulse wave, including the dicrotic notch. Hemodynamic forces are known to play an important role in vascular growth, remodeling, and integrity [39, 40]. Given that this first‐generation model focused only on successful microvessel culture and did not rigorously probe these variables, future work will focus on assessing how such hemodynamics influence salient features of our model system.

In conclusion, our study presents a significant advancement in the field of neurovascular research by successfully cultivating human brain microvessels within a microfluidic platform. The accurate replication of vascular architectures with barrier function could provide a valuable tool for studying neurovascular diseases and drug delivery across the BBB. One limitation of our model is the requirement for postmortem donor tissue, but the use of cryopreservation enables model scalability limited only by the number of banked tissue samples. In this regard, future work will focus on expanding the scope of molecular analyses of cellular identity and function across multiple donors, especially those lacking vascular pathologies. In addition, this work can likely be expanded to include other sources of neural cells (e.g. iPSC‐derived progenies) that will advance the physiological relevance of the model and its potential uses.

## Conflicts of Interest

The authors declare no conflicts of interest.

## Supporting information




**Supporting file**: adhm70760‐sup‐0001‐SuppMat.docx

## Data Availability

All designs and files from this work are available at: https://github.com/OGradyLab/NVU‐On‐A‐Chip.
