# Supplementary material for: Human Brain Vasculature‐on‐a‐Chip Model Constructed With Microvessels Isolated From Cryopreserved Postmortem Human Brain Tissue
Source: Adv Healthc Mater. 2026 Jan 12;15(13):e04167. doi: 10.1002/adhm.202504167 (PMC13058772; doi:10.1002/adhm.202504167)
Supplement: Supplementary file 1 — Supporting file: adhm70760‐sup‐0001‐SuppMat.docx [file ADHM-15-0-s001.docx]

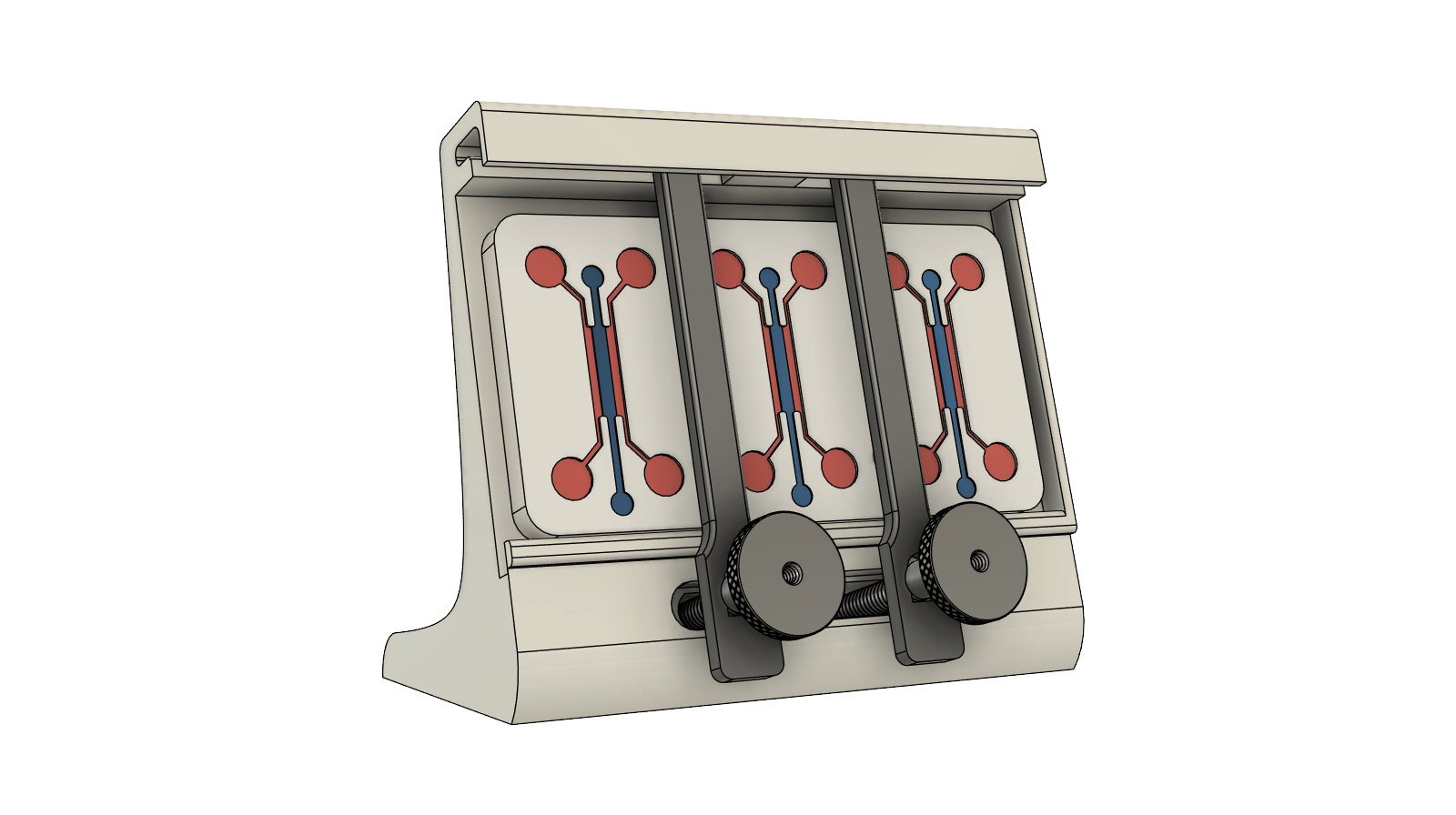
**Supplemental Figure 1: Custom microfluidic chip holder designed for hydrogel injection.** The holder positions the chip at a precise angle to ensure uniform filling of the central channel with hydrogel while preventing spillage into adjacent perfusion channels.

**Supplemental Figure 2: Immunofluorescence staining of microvessels cultured within the hydrogel for 4 days.** All images are from vessels isolated from the 73-year-old donor as described in the Methods section. **A)** Endothelial cells (PECAM1, green) and pericytes (NG2, red) forming intact microvessel structures. **B)** Smooth muscle cells (αSMA, red) surround the endothelium, confirming the presence of arteriole-like structures, while tight junctions (Claudin-5, gre
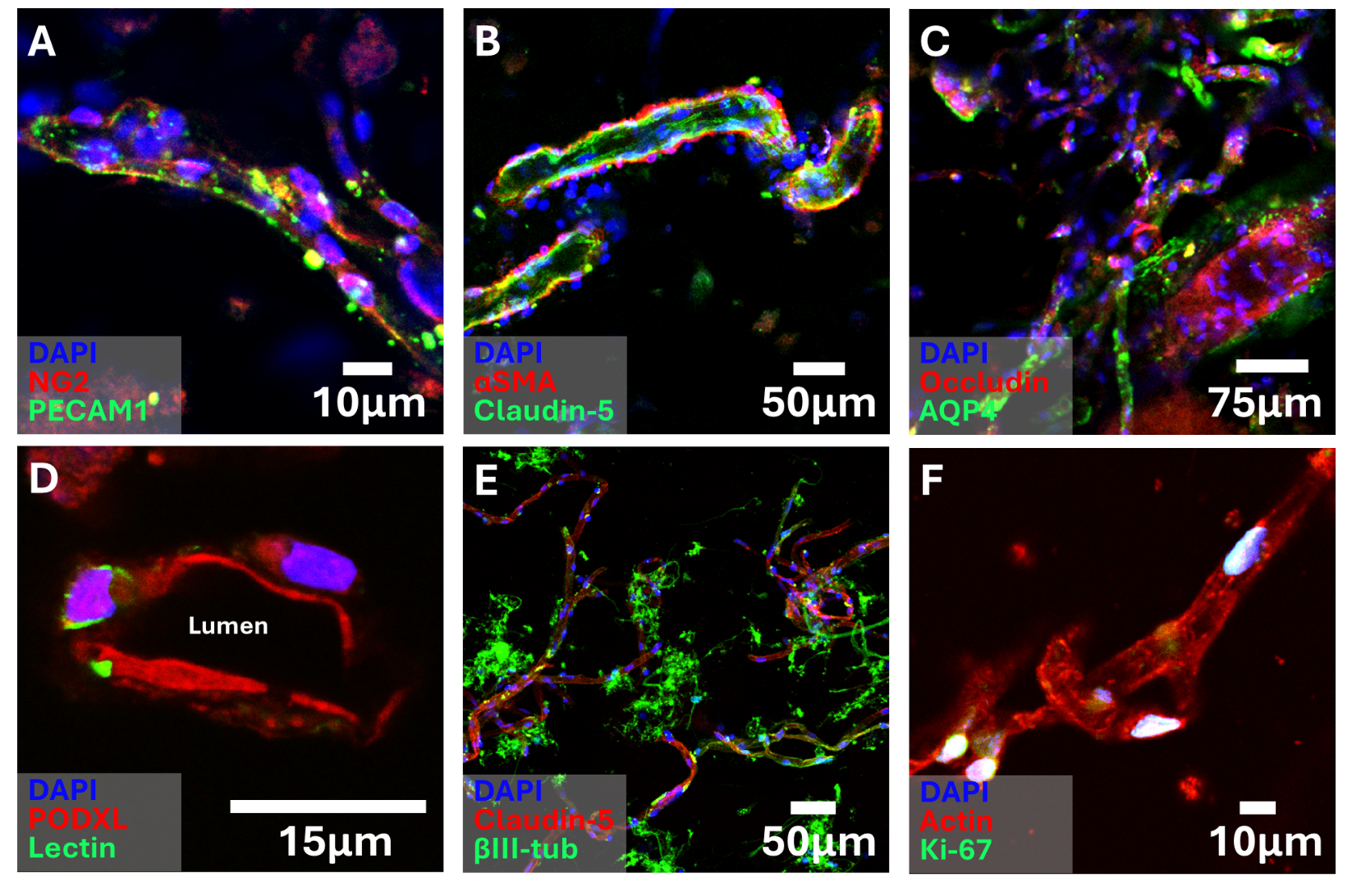
en) are clearly visible. **C)** Aquaporin-4 (AQP4, green) highlights astrocyte endfeet coverage around vessels. **D)** Lectin (green) binds to endothelial glycocalyx, confirming lumenized structures, with PODXL (red) marking the endothelial surface. **E)** βIII-tubulin+ neurons (red) are visible near Claudin-5+ (green) microvessels as potential remnants of the vessel isolation procedure. **F)** Proliferating cells are marked by Ki-67 (green), with actin (red) revealing vessel structure.


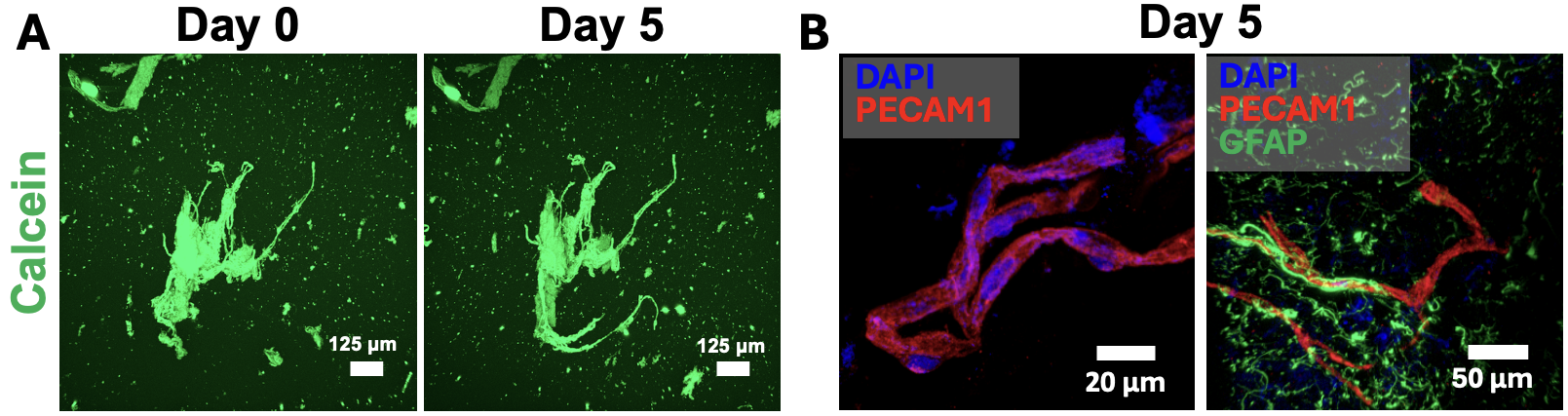


**Supplemental Figure 3: Confirmation of microvessel viability and identity from a second tissue donor.** All images are from vessels isolated from the 78-year-old donor as described in the Methods section. **A)** Calcein staining of viable microvessels immediately after embedding in the hydrogel (day 0) and 5 days of culture. Note the structure on the bottom of the tissue cluster that may be indicative of vascular proliferation or development. **B)** Representative images of endothelial cells (PECAM1) and astrocyte bodies (GFAP) at day 5 of culture.
